# Individual preferences modulate incentive values: Evidence from functional MRI

**DOI:** 10.1186/1744-9081-4-55

**Published:** 2008-11-25

**Authors:** Susan Koeneke, Andreas F Pedroni, Anja Dieckmann, Volker Bosch, Lutz Jäncke

**Affiliations:** 1 University of Zurich, Institute of Psychology, Division Neuropsychology, Switzerland; 2 GfK Association, Basic Research, Nuremberg, Germany

## Abstract

**Background:**

In most studies on human reward processing, reward intensity has been manipulated on an objective scale (e.g., varying monetary value). Everyday experience, however, teaches us that objectively equivalent rewards may differ substantially in their subjective incentive values. One factor influencing incentive value in humans is branding. The current study explores the hypothesis that individual brand preferences modulate activity in reward areas similarly to objectively measurable differences in reward intensity.

**Methods:**

A wheel-of-fortune game comprising an anticipation phase and a subsequent outcome evaluation phase was implemented. Inside a 3 Tesla MRI scanner, 19 participants played for chocolate bars of three different brands that differed in subjective attractiveness.

**Results:**

Parametrical analysis of the obtained fMRI data demonstrated that the level of activity in anatomically distinct neural networks was linearly associated with the subjective preference hierarchy of the brands played for. During the anticipation phases, preference-dependent neural activity has been registered in premotor areas, insular cortex, orbitofrontal cortex, and in the midbrain. During the outcome phases, neural activity in the caudate nucleus, precuneus, lingual gyrus, cerebellum, and in the pallidum was influenced by individual preference.

**Conclusion:**

Our results suggest a graded effect of differently preferred brands onto the incentive value of objectively equivalent rewards. Regarding the anticipation phase, the results reflect an intensified state of *wanting *that facilitates action preparation when the participants play for their favorite brand. This mechanism may underlie approach behavior in real-life choice situations.

## Background

What counts as reward differs substantially depending on individual preferences. Branding can elicit robust differences in preferences for consumer products despite their highly similar appearance and may therefore provide an ideal example of truly subjective preference in that it is largely independent of objective stimulus characteristics. Indeed, branding can be viewed as the assignment of value and meaning to often quite mundane and interchangeable products [e.g., [1]]. Current theories of reward processing have paid increasing attention to such cultural influences on choice behavior. Our aim is to expand these theories by examining the modulatory impact of subjective brand preferences on neural activity. Furthermore, the external validity of our findings is enhanced by the high relevance of brands in everyday life.

Most previous research on the neural representation of reward has focused on the manipulation of reward according to an objectively quantifiable scale without therefore having to consider individual differences in preferences. There is no doubt that people have a general preference for larger rather than smaller amounts of money. Many human imaging studies have made explicit use of various degrees of monetary incentive value as means to manipulate reward intensity, and have reported several neural regions that adapt their activity according to the changes in reward intensity. O'Doherty et al., for example, reported stronger recruitment of the medial orbitofrontal cortex (OFC) upon gaining higher compared with smaller amounts of money in a two-alternative choice task [2]. In another study, Breiter et al. identified the sublenticular extended amygdala (SLEA), the Nucleus accumbens (NAcc) and the hypothalamus as showing a scaled neural response in correspondence with the value of received monetary gain [3]. In addition, neural activation patterns in the SLEA and in the OFC reflected the value of the potential rewards in the period in which participants anticipated the outcome. A later study by Knutson et al. found a dissociation of neural circuits involved in different aspects of reward: While the ventral striatum (incl. NAcc) was strongly active during the anticipation of monetary reward, the mesial prefrontal cortex (PFC), the parietal cortex and the posterior cingulum were active following feedback to the participants of having successfully obtained a reward [4]. During reward anticipation, the NAcc activity was positively correlated with the magnitude of the monetary reward.

However, even the rewarding value of money may be influenced by context effects. Counterfactual reasoning, for example, refers to people's tendency to compare their choices with the outcome of alternative courses of action. Winning Sfr. 5 in gambling most certainly evokes some degree of satisfaction, whereas winning Sfr. 5 while knowing that one could have won Sfr. 10 had one chosen differently evokes regret or disappointment [5]. The neural underpinnings of this effect have recently been investigated [6-8]. The concept of delayed discounting, concerning the point in time when a reward is delivered, is a further example of the effect of contextual information on the perceived value of a certain reward [9-12]. Animal studies strengthen this finding [13,14]. Counterfactual reasoning and delayed discounting, however, reflect general effects that hold across subjects, meaning that the contextual information has a similar impact on each subject.

Food, on the other hand, might be a universal primary reinforcer but people greatly differ in their taste preferences. Similarly, interindividual variance in the attractiveness of a reward also characterizes branded consumer goods. The neural representation of brand preferences has recently received considerable attention. McClure, for instance, reported that participants show stronger hemodynamic responses in reward-related brain regions (dorsolateral prefrontal cortex (DLPFC), hippocampus, midbrain) when receiving a small amount of a soft drink pre-cued by a picture of a Coca-Cola can rather than by a circle of light or a picture of a Pepsi can [15]. Schaefer and Rotte reported stronger activity in the medial prefrontal cortex (MPFC) and precuneus when participants were presented with logos of luxury and sports car brands compared with pragmatic, more economic car brands [16]. In a study by Deppe et al., participants were asked to imagine choosing between pairs of brands [17]. The authors reported reduced neural activity in regions associated with working memory and reasoning, and increased neural activity in regions related to emotion processing when presenting the most popular brand in terms of the market share as compared with less popular brands. Based on their findings, the authors postulate a *winner-take-all *effect of a person's favorite brand on neural activation, an effect that would partially contradict the graded response to different amounts of monetary rewards.

To our knowledge, however, none of the available studies on brand preferences used participants' stated preferences as a means to specifically varying the subjectively perceived attractiveness of the presented brands. Furthermore, previous brain imaging studies of brand preferences did not clearly differentiate between the period of reward anticipation and that of reward receipt. This distinction between anticipatory (*wanting*) and evaluative (*liking*) components has already been proposed by Berridge on the basis of animal studies [18], and evidence from human studies using monetary reward supports this concept [3,19]. Thus, the available studies on brand preferences may have confounded motivational with evaluative components of reward processing. Finally, the use of more than two preference categories is a necessary precondition to unequivocally determining any modulatory influence of brand preference on neural activity patterns. It may well be that, similar to monetary rewards, brand-associated neural activity increases monotonically with the strength of the individual preference for a particular brand.

To address these issues, we developed a wheel-of-fortune game that allowed for the differentiation between an anticipation period (spinning of the wheel; wishing for a positive outcome) and an outcome period (processing the game outcome). Chocolate bars of three different brands could be won. By using chocolate bars as rewarding stimuli we introduced a product category with relatively homogeneous pricing to avoid the coupling of reward intensity with monetary value, which may be neurally processed in a different way. Established market research instruments were used prior to the fMRI experiment to determine participants' individual brand preferences. Based on the results of these instruments, brands that differed in subjective attractiveness were selected individually for each participant and used as stimuli in an fMRI experiment. During the experiment, brands were represented by their logos. However, real chocolate bars were given to the participants after the experiment.

The primary aim of our study was to explore whether there are neural structures that modulate their activation according to the subjective preferences for the chocolate bar brands that the participants played for (e.g., higher activity in case of more preferred compared to less preferred chocolate brands). Additionally, the design allowed for investigating the suggested dissociation between an anticipatory reward component (game outcome unknown, wanting) and an evaluative reward component (evaluation of game outcome, liking). This dissociation is important for understanding buying behavior, since *anticipation *and *evaluation *are associated with different facets of a brand: (a) motivational, action-relevant characteristics, and (b) emotional or cognitive evaluative aspects.

## Methods

### Participants

Nineteen healthy female adult voluntary participants (mean age of 24.05 ± 2.63) were recruited from the University of Zurich and ETH Zurich, Switzerland. Participants were selected based on a two-stage selection procedure. At the first stage, a paper and pencil questionnaire was distributed to students in different courses of the Psychology Department of the University of Zurich. Ninety-eight students completed the questionnaire. Of those, thirty-one respondents who indicated that they (a) ate chocolate at least from time to time, (b) cared about chocolate, (c) cared about brands when it came to chocolate and who expressed differentiated brand preferences in a constant sum point allocation "chip game" between different chocolate brands, were invited to the second round. Given that the majority of the participants who passed this first phase were female, we decided to restrict the study to women. However, we do not expect gender differences in the neural representation of rewards differing in subjective attractiveness. Twenty-seven of the pre-selected participants accepted the invitation and filled out a second, computer-based questionnaire that aimed at measuring individual brand preferences in more detail with a choice-based procedure (the GfK Price Challenger, GPC [20]) and, again, with a constant sum chip game. Of those, twenty respondents who expressed preferences that were consistent across the two measures and widely dispersed to allow for clear brand differentiation were finally invited to the fMRI study. One participant dropped out for private reasons. The remaining nineteen participants gave informed consent approved by the local ethics committee. Participation was compensated with 50.00 sFr and the amount of chocolate bars won.

### Task design

Participants played a virtual wheel-of-fortune game presented via a video projector onto a translucent screen that participants viewed inside the scanner via a mirror. The experiment consisted of four runs with 30 trials each. Routinely, individual T1-weighted anatomic brain images were recorded before the actual experimental sessions started. The total scanning time was approximately 50 minutes.

Before being scanned, participants were carefully informed with respect to the MRI/fMRI method. Following this, each participant had to (1) complete a questionnaire that checked for individual MR-suitability and (2) to give his/her written informed consent. Then, participants were requested to read a short instruction manual, which explained the procedures of the experiment, and played two trials of the wheel-of-fortune game outside the scanner in order to make sure that they had understood the task.

The experiment had a 3 × 2 × 2 factorial design: Participants played for three different chocolate brands (1^st ^factor). These brands were selected based on the preference data gathered in the second stage of the selection procedure. For each participant, her favorite and her least preferred yet still acceptable brand were selected, as well as one intermediate brand that ranked between the top and the bottom brand. There were two types of trials (2^nd ^factor), winning trials and losing trials, with two possible outcomes, respectively (3^rd ^factor): In winning trials participants either won or did not win a chocolate bar; in losing trials, already won chocolate bars were either lost or not lost. The main focus of our study was on the hemodynamic responses to winning trials, that is, to positive anticipation and outcomes. We implemented separate losing trials rather than combining winning and loosing in one trial (win a chocolate bar vs. loose a chocolate bar) in order to detach negative, apprehensive processes that might predominate in some participants from more cheerful positive expectancy. There is recent empirical evidence that participants anticipate emotional events of unknown valence to be negative or unpleasant [21]. By separating the anticipation of positive from the anticipation of negative outcomes we circumvented this potential problem.

Participants were randomly assigned to one of six different pseudorandom trial sequences. In each trial, the chance of winning or losing a chocolate bar was approximately fifty percent. Also the brands the participants played for were pseudo-randomly distributed to ensure enough trials of every possible combination (brand × trial type × outcome) for the analysis.

**Figure 1:**
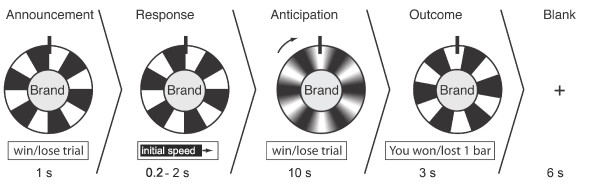
Experimental design of the wheel-of-fortune game.

One trial consisted of an announcement phase (1 sec.), a response phase (0.2 – 2 sec), an anticipation phase (10 sec.), an outcome phase (3 sec.), and a blank screen with a fixation cross (6 sec.; see Figure 1). In the announcement phase the brand logo was presented in the middle of a wheel of fortune with six colored (green for wining trials, red for losing trials) and six black fields. The colors indicated the trial type (winning trial vs. losing trial). During the response phase, participants could control the entry speed of the rotation of the wheel of fortune by pressing a button early or late within the time window. This was implemented to give participants the feeling of being actively involved in the game. Additionally, the variable response latency (200 ms – 2000 ms) induced a dephasing of stimulus onsets with respect to scan onsets to optimize sampling of the hemodynamic response. The entry speed did not affect the (pseudo-randomized) outcome of the prior anticipation phase. The anticipation phase started with the wheel of fortune rotating at the selected entry speed, slowing down to halt after 10 seconds. The ensuing outcome phase started after the wheel had stopped. The outcome was indicated by the field that came to a halt under a pin at the top of the wheel and it was also indicated in a text box (i.e., "You have won/lost 1/0 chocolate bars"). To ensure that the fMRI signal could level back to a task-unspecific baseline, a blank screen with a fixation cross was presented for six seconds before the next trial started.

At the beginning of the experiment, each participant started with an account of three chocolate bars of each brand. It had been made clear to participants during the instructions prior to scanning that all major tastes of the brands they played for were available to choose from (e.g., dark chocolate, milk chocolate, hazelnut). Thus, participants did not have to fear that they would end up with tastes they did not like. After each of the four runs the number of chocolate bars was accounted and the balance was visually presented. This balance was transferred to the next run. After the experiment, participants received the total number of chocolate bars won (on average 8.83 chocolate bars), thus ensuring that the wheel-of-fortune game offered real incentives. Finally, after the four runs were finished, the participants were paid 50.00 sFr, given the won chocolate bars in the taste variants of their choice, and dismissed.

Trials in which participants missed starting the wheel of fortune (i.e., did not press the button within two seconds) were regarded as no-interest trials and excluded from the statistical analyses. The total number of missed trials across all participants was 8, with a maximum of two lost trials for two of the participants.

### Functional imaging

A Philips Intera 3T whole-body MR unit (Philips Medical Systems, Best, The Netherlands) equipped with an eight-channel Philips SENSE head coil was used to acquire magnetic resonance images at the University Hospital Zurich. Anatomical images of the whole brain were obtained by using a T1-weighted three-dimensional, spoiled, gradient echo pulse sequence (repetition time (TR) = 20 ms, echo time (TE) = 2.30 ms, flip angle = 20°, field of view (FOV) = 220 mm, acquisition matrix = 224 × 224, voxel size = 1.00 × 1.00 × 0.75 mm, 180 slices, slice thickness = 0.75 mm). Functional data for the behavioral tasks were obtained from 280 whole-head scans per run (1120 for 4 runs) using a Sensitivity Encoded (SENSE) [22] single-shot echoplanar imaging technique (TR = 2500 ms, TE = 35 ms, flip angle = 78°, FOV = 220 mm, acquisition matrix = 80 × 80, 33 transverse slices, voxel size = 1.72 × 1.72 × 4.00 mm).

### Data analysis

Artifact minimization and MRI data analysis were performed using MATLAB 2006b (Mathworks Inc., Natick, Massachusetts, USA), and the SPM5 software package (Institute of Neurology, London, UK). The first three images were discarded to allow for steady-state magnetization. All images were realigned to the first image of the first run, spatially normalized into standard stereotactic MNI-space (EPI template provided by the Montreal Neurological Institute), interpolated to a voxel size of 2.00 × 2.00 × 2.00 mm and spatially smoothed using a 8-mm full-with-at-half-maximum Gaussian kernel.

Activated voxels were identified by the general linear model approach, implemented in SPM5. At the first level of analysis, we adopted a parametric analysis according to Büchel et al. [23]. After highpass-filtering (cut-off 128 s), an individual statistical model was computed for each participant with separate regressors for the response phase (modeled as events), for the anticipation phase of winning and losing trials (each modeled as epochs of 10 s), and for each possible outcome type (won winning trial, not-won winning trial, lost losing trial, not-lost losing trial, modeled as epochs of 3 s). All regressors were convolved with SPM's canonical difference of gamma hemodynamic response function. The maximal cross-correlation between regressors was on average ρ = 0.156 (SD = 0.033) across all subjects.

Given that the main purpose of the analysis was to identify regions whose hemodynamic response monotonically increased or decreased with individual brand ranking, the ranks of the brands in the individual preference hierarchy were included in the model as modulatory parameters (i.e., 3, 2, 1, from the most to the least preferred brand). Linear contrasts of the first-order terms against a baseline (6 seconds rest epoch, blank screen with fixation cross) were performed. This was applied to the anticipation phases of winning trials and losing trials, the outcome phases of winning trials that were won and not won, and the outcome phases of losing trials that were not lost and lost (contrasts are indicated by ** _1_, e.g., WA_1_). To additionally obtain results of the main effect of the task, individual baseline contrasts were performed using the zero^th ^order regressor of the respective conditions (contrasts are indicated by ** _0_, e.g., WA_0_). A complete list of all experimental conditions is given in table 1.

**Table 1:** List of experimental conditions.

Trial type:	Phase:	Outcome:	Abbreviation:
Winning	Anticipation	Won & not won	WA
Winning	Outcome	Won	WOW
Winning	Outcome	Not won	WOnW
Losing	Anticipation	Lost & not lost	LA
Losing	Outcome	Lost	LOL
Losing	Outcome	Not lost	LOnL

To permit population-level inferences, maps of contrast coefficients for each of the first level contrasts were collectively submitted to one-sample *t*-tests against the null hypothesis of no activation, while controlling for random effects. Given that the outcome phase immediately followed the anticipation phase yields the possibility that clusters of activation found in the outcome phase are also due to continuing activity elicited during the anticipation phase. Taking this possible confound into account, we additionally reduced the search area for activations in the outcome phase (WOW_1_, WOnW_1_, LOL_1 _LOnL_1_) to the areas activated by the preceding anticipation phase (WA_1_, LA_1_). No clusters of activation remained.

To explore the full range of effects in the data, voxels surviving significance thresholding at *p *< .001, uncorrected for multiple comparisons with a spatial extent threshold at k = 10 voxel were reported. For specific regions a priori hypotheses could be derived from findings of prior studies using reward paradigms [19,24-26]. Small volume corrections (SVCs) were used for these regions to correct the false positive error probability for the number of comparisons made within each region. SVCs were applied with a sphere of 8 mm, chosen to be equal to the spatial smoothing kernel [27-29]. Peaks surviving *p *< .05 family-wise error (FWE) correction were considered significant. The cluster locations were indicated by the coordinates of the voxel at the local cluster maximum and labeled using the automated anatomical labeling (AAL) toolbox [30]. Cluster locations that were not identified with the AAL toolbox were manually labeled with reference to the Harvard-Oxford subcortical structural atlas. By overlaying the statistical parametric maps on an averaged and normalized structural (T1) image of all subjects, we ensured that the cluster locations were within the reported neuronal structures.

## Results

The main focus of our study was placed on brain regions in which neuronal responses increase or decrease monotonically with increasing brand preference during the anticipation phase preceding winning trials (WA) and the outcome phase following gains in winning trials (WOW). This represents the first-order term in the parametric analysis. We also included losing trials into our experiment to balance the amount of gained rewards and to dissociate gain from loss phases (see methods section). For descriptive purposes, we additionally conducted first-order parametrical analyses of anticipation phases of losing trials (LA_1_) and outcome phases of lost losing trials (LOL_1_) [Additional file 1]. For outcomes with no effect on gaining or losing chocolate bars (WOnW_1 _and LOnL_1_), no significant preference-modulated clusters were located (*p *< .001 for multiple comparisons). Thus, the reported findings refer to expectations and outcomes of rewards (chocolate bars) rather than to an unspecific effect of brand logo presentation.

### Main effects of task

The effects of the zero^th ^order term of the parametric analysis (main effect of the tasks) were not of interest for the current study question. For the sake of completeness the corresponding results of WA_0 _and WOW_0 _are listed in the supplement [Additional file 2].

### Anticipation phase of winning trials

The contrasts of the first order parametric modulation of the anticipation phase of winning trials (WA_1_) revealed several brain areas that showed linearly *increasing *hemodynamic responses with higher subjective preference: Left caudal premotor area, right rostral premotor area, right lateral orbitofrontal cortex reaching into the anterior insula, right posterior superior temporal sulcus/anterolateral intraparietal sulcus, and the dopaminergic midbrain.

**Figure 2:**
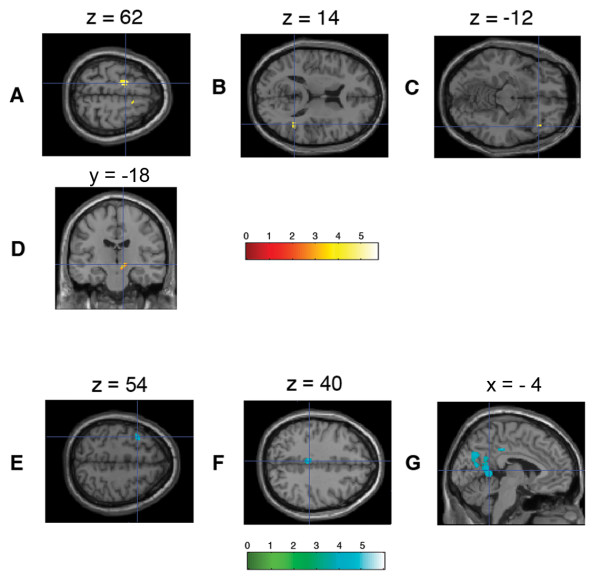
**Brain regions showing preference-modulated activation during the anticipation phase of winning trials**. (A) Bilateral mesial premotor/supplementary motor area showing most powerful activations, (B) right superior temporal sulcus, and (C) right anterior insula/lateral orbitofrontal cortex with significant activation patterns at uncorrected level of *p *< .001 with clusters with more than 10 voxels. (D) A cluster of midbrain activation was found at a close to significant level after small volume correction at threshold level *p *< .01. Neural activity in brain regions negatively correlated with the brand preference (i.e., showing less activation for more preferred brands) during the anticipation phase: (E) left frontal middle gyrus, (F) left posterior cingulate cortex, (G) left precuneus.

Clusters of voxels showing a linear *decrease *in neural activity with higher subjective preferences (WA_1_) were located in the left middle frontal gyrus, left middle cingulate cortex, bilateral precuneus, left calcarine sulcus, left angular gyrus, left lingual gyrus, left fusiform gyrus and right middle cerebellum (Figure 2, Table 2).

**Table 2:** Clusters showing preference-dependent activation during the anticipation phase.

Neural activity in regions	Right/Left	Cluster Size (Voxels)	Coordinates			*t*-value
			X	Y	Z	
increasing linearly with subjective preference:						
**Caudal premotor area**	**L**	**99**	**-16**	**-8**	**62**	**5.89**
**Rostral premotor area**	**R**	**13**	**16**	**4**	**68**	**3.86**
**Lateral orbitofrontal cortex/anterior insula**	**R**	**15**	**44**	**28**	**-12**	**3.97**
Posterior superior temporal sulcus/anterolateral intraparietal sulcus	R	11	42	-48	14	4.18
**Dopaminergic midbrain (substantia nigra)**	**R**	**33**	**10**	**-18**	**-6**	**4.01**
decreasing linearly with subjective preference:						
Middle frontal gyrus	L	71	-36	12	60	6.02
Posterior cingulate cortex	L	32	-4	-30	40	4.85
Posterior cingulate cortex	L	38	-16	-50	36	4.36
Precuneus	L	443	-4	-50	8	5.67
Precuneus	R		6	-52	14	5.23
Precuneus	L		-2	-58	26	3.84
Precuneus	L	173	-2	-72	34	4.66
Calcarine sulcus	L		-4	-70	16	4.08
Middle occipital cortex	L	88	-42	-76	34	4.74
Angular gyrus	L		-52	-74	26	4.78
Lingual gyrus	L	35	-8	-80	-8	4.34
Lingual gyrus	L		-16	-82	-6	3.92
Fusiform gyrus	L	13	-26	-42	-14	4.28
Middle cerebellum 10	R	15	28	-36	-40	4.65

All clusters show a probability of error of *p *< .001 uncorrected for whole brain multiple comparisons. The coordinates and *t*-values are at the peak voxels in each cluster (coordinates refer to MNI-space). Clusters written in bold letters are within a priori hypothesized regions and remained significant after family-wise error (FWE, p < 0.05) correction applied for small volumes (sphere with 8 mm radius).

### Outcome phase of won winning trials

**Figure 3:**
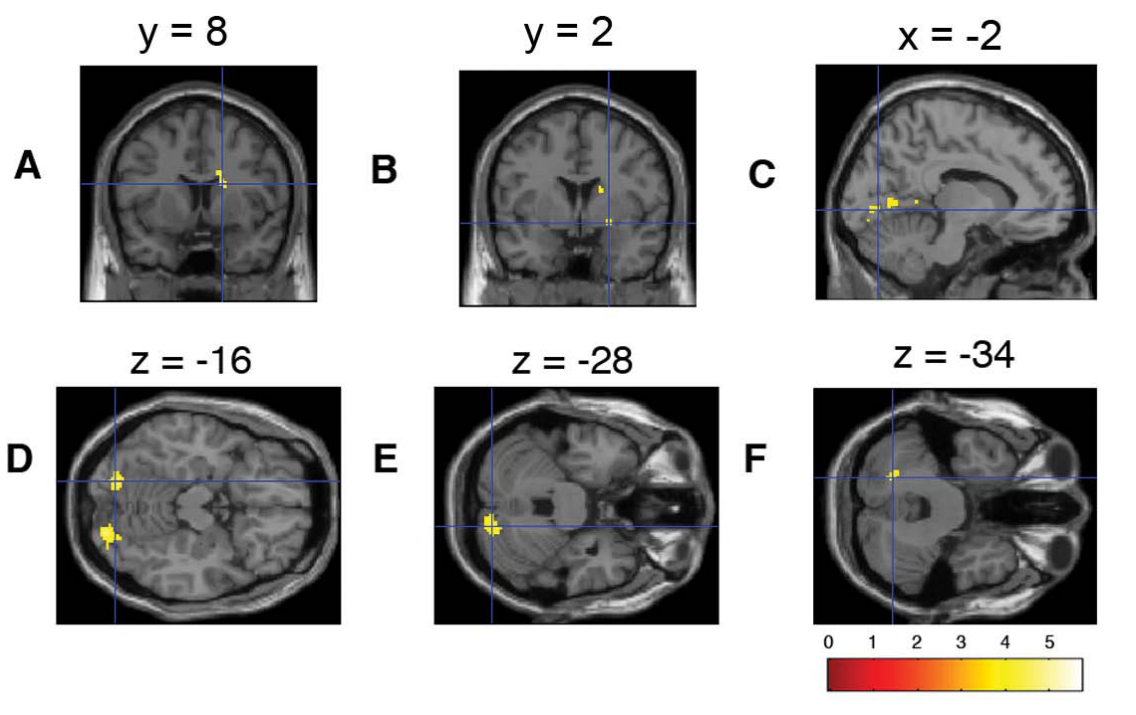
**Brain regions showing preference-modulated activation during the outcome phase of won winning trials**. (A) Caudate nucleus, (B) pallidum, (C)/(D) lingual gyrus, (E)/(F) cerebellum crus 1.

In the outcome phase of won winning trials, clusters of voxels in the following regions increased their hemodynamic response linearly with higher subjective preference for the reward (WOW_1_): The right precuneus, right supramarginal gyrus, left and right lingual gyrus, left posterior cingulum, right caudate nucleus, right superior temporal sulcus, right postcentral gyrus, right and left cerebellum including the vermis, left middle temporal gyrus, left superior occipital areas, right frontal inferior operculum, right superior frontal area, left angular gyrus and right ventral pallidum (Figure 3, Table 3).

**Table 3:** Clusters showing preference-dependent activation during the outcome phase.

Neural activity of regions increasing linearly with subjective preference:	Right/Left	Cluster Size (Voxels)	Coordinates			*t*-value
			X	Y	Z	
**Caudate nucleus**	**R**	**51**	**18**	**8**	**18**	**5.19**
**Caudate nucleus**	**R**		**16**	**2**	**26**	**4.92**
**Ventral pallidum**	**R**	**82**	**24**	**2**	**-8**	**4.28**
Precuneus	R	200	6	-46	6	5.88
Posterior cingulum	L		-2	-42	8	5.21
Vermis	L/R		0	-54	-4	4.24
Precuneus	R	24	16	-60	40	5.25
Lingual gyrus	L	187	-20	-72	-4	5.29
Lingual gyrus	L		-14	-82	-12	4.89
Lingual gyrus	L		-12	-80	-2	4.68
Superior occipital	L	16	-14	-96	20	4.47
Lingual gyrus	L	16	-14	-56	0	3.90
Lingual gyrus	L	29	-6	-66	4	4.03
Lingual gyrus	R	74	22	-90	-16	4.82
Inferior occipital gyrus	R		34	-22	-16	3.74
Lingual gyrus	R	35	22	-52	-2	4.40
Lingual gyrus	R		14	-50	-4	4.26
Cerebellum crus 1	R	140	16	-82	-28	4.62
Cerebellum crus 1	R		6	-20	-22	4.34
Cerebellum crus 1	L	20	-22	-66	-34	4.22
Superior temporal gyrus	R	19	52	-26	16	4.90
Supramarginal gyrus	R	62	42	-42	22	5.57
Supramarginal gyrus	R	13	46	-28	28	4.49
Middle temporal gyrus	L	13	-38	-56	16	4.55
Angluar gyrus	L		-42	-52	22	3.98
Postcentral gyrus	R	13	38	-30	54	4.86
Frontal inferior gyrus, triangular part	R	14	28	16	20	4.16
Superior frontal gyrus	R	12	18	28	40	4.15

All clusters show a probability of error of *p *< .001 uncorrected for whole-brain multiple comparisons. The coordinates and *t*-values are at the peak voxels in each cluster (coordinates refer to MNI-space). Clusters written in bold letters are within a priori hypothesized regions and remained significant after family-wise error (FWE, p < 0.05) correction applied for small volumes (sphere with 8 mm radius).

The analysis revealed no significant clusters of voxels which show a linear decrease in activity with increasing subjective preference (WOW_1_).

## Discussion

The anticipation of acquiring desired objects plays an essential role in everyday life. There are clear interindividual differences in the preferences for choice alternatives, be it in connection with fashion, food, or cars. However, it is unclear to what extent subjectively defined preference levels (e.g., most preferred brand) modulate activation in brain regions that are typically involved in reward processing. Therefore, the purpose of the present study was to investigate brain areas that are sensitive to subjective reward intensity. For this purpose, we evaluated the neural activation patterns associated with the expectation and evaluation of receiving desired compared to less desired objects. A further aim of this study was to examine whether the modulation of neural activity by the intensity of brand attractiveness was evident in distinct neural networks during the anticipation of the desired objects and during the evaluation of the receipt of these objects. Using a wheel-of-fortune game, we found that during anticipation the hemodynamic responses in the premotor cortex, the lateral orbitofrontal cortex, the insula, and the dopaminergic midbrain are linearly correlated with the subjective preference of a desired object. Activation in these areas was strongest while the participants expected to win the most desired object. In addition, the hemodynamic responses in the left middle frontal gyrus, posterior cingulate cortex and several extrastriate visual areas were negatively correlated with the expectation to win a desired object. In the ensuing outcome phase, while participants evaluated the positive game outcome, a distinct neural network commonly associated with attentional processes, sympathetic arousal, and cognitive-emotional evaluation of rewards showed preference-modulated activity.

### Anticipation phase of winning trials

The most striking finding of our study is a linear increase in hemodynamic responses in the left caudal and rostral premotor cortex the more participants desired to win a chocolate bar. Previous studies also found reward-dependent activation in premotor areas. It was, for example, reported that premotor regions become more active with increasing monetary reward in a target detection task [26]. Also, in non-human primates, dorsal but also lateral prefrontal regions including the premotor cortex were rendered active while expecting rewards [31]. Furthermore, Roesch and Olson reported increasing activity in premotor neurons in macaque brains dependent on the value of a predicted reward [32]. In contrast to these studies, reward delivery in our study did not depend on an instrumental motor action (e.g., grasping a reward). Thus, a simple motor preparation account is not sufficient to explain our finding. We interpret the increase in bilateral premotor activity as an increased state of motor preparedness, which may facilitate approaching behavior. Modulation of motor preparedness by different values of subjective brand preferences may occur automatically due to action-inducing characteristics of such incentive stimuli. In a low-involvement buying situation increased premotor activity could already be sufficient to "tip the scales" so that a person snatches at one product without making a deliberate decision to do so.

The reduced hemodynamic responses in the left MFG in anticipation of winning a more preferred chocolate bar may reflect the functional antagonist to the increased premotor activity. In a meta-analytic study, Rubia et al. report that this area (besides others) is activated in several Go/No-Go tasks – a task demanding high-level cognitive functions of decision-making, response selection and response inhibition [33]. When playing for a more preferred chocolate brand in our study, such cognitive response control may be reduced. Support for this idea comes from Deppe et al. who report decreased neural activity in the left hemispheric middle frontal gyrus when participants imagined making binary decisions between a target brand, which was the market leader, and another (less popular) brand, as compared with choices between two less popular brands [17]. In another recent study, Schaefer and Rotte found reduced activation in a right hemispheric homologue when participants saw attractive car brands compared to less attractive car brands [34]. Both research groups concluded that rational thinking might be reduced when confronted with favored brands.

In summary, the pattern of activity in the above mentioned neural network indicates an increased state of motivation for motor action (e.g., facilitating approaching behavior). But what inherent properties of an object make it more desirable (so that it will be approached more frequently) than others? The increased hemodynamic responses in the right anterior insula/lateral orbitofrontal cortex when playing for preferred chocolate brands may signal enhanced somatic arousal associated with a favorite reward. Supporting this idea, the right insula plays a prominent role in the somatic-marker hypothesis [35]. According to this hypothesis, insular activation provides a neural substrate of emotional feeling states arising from automatic somatosensory responses, making them available to cortical processing and conscious awareness. In line with this idea, Critchley and colleagues found right hemispheric activation in anterior insular and orbitofrontal regions associated with sympathetic arousal in a reward-related decision-making task [36]. The authors suggested that these two regions are modulated by changes in peripheral somatic states and involved in the flexible representation of reinforcement [37].

We discovered preference-modulated hemodynamic responses in mesolimbic regions in the right midbrain. Dopaminergic neurons in the midbrain reflect the incentive or motivational value of a future reward and are associated with a subjective state of wanting [25,38]. Additionally, studies with non-human primates demonstrated increased firing rates in dopaminergic midbrain neurons during the anticipation of rewards after associations between predictive cues and reinforcers have been learned [39]. In the case of the chocolate brand logos that we used, the association between the predictive cues (i.e., the logos) and reinforcers (e.g., delicious chocolate) has likely been established by previous learning experiences of our participants.

The cluster of preference-modulated activity in the right anterolateral intraparietal region, which extends into the superior temporal sulcus (STS) probably reflects the process of inferring from the motion of the wheel whether the trial will be won or not; the higher the incentive value of the reward, the more relevant is this prediction. In previous studies, increased activity in this area was assumed to reflect action-outcome prediction through observation [40,41]. Furthermore, the anatomical proximity to the parietal cortex, which has been found to be involved in visuo-spatial processing [42], underpins the notion that this area could be involved in the processing of spatial contiguity between current position and desired outcome position.

The negatively correlated neural activity (lower hemodynamic response while anticipating more desired objects) found in regions encompassing the posterior cingulate gyrus, precuneus, lingual gyrus, fusiform gyrus and cerebellum may be due to task-induced deactivation (TID). TID refers to a relative decrease in regional activity, as measured by blood flow or the blood oxygenation level dependent (BOLD) signal, during an active task compared to a "resting" baseline [43]. We believe that the decrease in the BOLD signal in the above mentioned neural structures refers to a higher externally cued cognitive involvement in the anticipation phase for more preferred brands compared to less preferred brands, resulting in a higher suppression of internally generated information processing. The study of McKiernan showed that TID increased with task processing demands [43]. TID often occurs in the posterior cingulate cortex extending dorsally into the precuneus [44,45], but also in the precuneus and fusiform gyrus [43], and was repeatedly shown to be of higher magnitude in the left cortical hemisphere [44-46].

In summary, hemodynamic responses increased in areas associated with motor preparation, emotional tagging of stimuli, reward expectation and spatial attention in the anticipation phase of the wheel-of-fortune game while playing for a more desired item. Conversely, neural activity in structures involved in stop inhibition of motor responses and internal information processing linearly decreased. While expecting the outcome, participants encountered an increased state of wanting (dopaminergic midbrain↑), external information processing (TID areas↓) and emotional tagging of the incentive stimulus (left anterior insula↑), leading to a state of facilitated action induction (bilateral premotor cortex↑, middle frontal gyrus↓).

### Outcome phase of won winning trials

In the time window after the participants saw the final outcome position of the wheel of fortune, preference-modulated activations were found in the caudate nucleus, precuneus, lingual gyrus, cerebellum, and, to a lesser extent, in the pallidum. Our results seem to reflect preference-dependent modulation of attentional processes, sympathetic arousal, and of cognitive-emotional evaluation of the reward value.

When participants were "rewarded" with more preferred chocolate bars, we found increased activity in the right caudate nucleus, traditionally seen as a "motor" region. Findings of Haruno et al. suggest, however, that the caudate nucleus is strongly involved in reward based behavioral learning [47]. It has further been shown in monkeys [31,48] and rats [49] that part of caudate-putamen neurons respond to food and drink reward stimuli in a manner similar to dopaminergic or ventral striatal neurons.

The ventral pallidum (VP) has been suggested to represent a central relay station for the distributed brain circuit of core liking [18,50], as well as a potential relay station to cortical systems of conscious pleasure [50]. Neurons in the VP are assumed to track the hedonic value of rewarding and appealing stimuli [51,52]. Besides the activation of "liking" structures via more primary taste rewards and sexual or competitive arousal, it has been shown that more abstract pleasures like monetary rewards also increase activity in the VP [19].

In the outcome phase many occipito-parietal regions, like the precuneus and parts of the lingual gyrus where found to be more active when winning a more preferred chocolate bar. We interpret this assembly of activations as a neural representation of top-down controlled visual attention. Playing for more preferred compared to less preferred chocolate bars is likely associated with a higher interest in the game outcome, which might cause a stronger attention focus on the outcome situation (visual perception and processing of the outcome). A cue for top-down attentional orientation in the visual field could be provided by an early tagging of emotional stimuli as Schupp et al. inferred from recent EEG studies [53-55].

We found brand-preference-modulated activity also in the cerebellum, namely in the vermis and right-sided crus 1. In addition to its predominant role in motor functions, it has been shown that the cerebellum is involved in higher cognitive and emotional processes [56]. The cerebellum is also an important component of autonomic control functioning. In line with this idea, Critchley et al. found distributed cerebellar activations similar to ours when participants experienced states of arousal [57]. Regarding our study, we can only speculate that some heightened state of arousal was induced by winning a highly desired compared to a less desired chocolate bar.

The interpretation of the activations in the superior temporal sulcus, middle temporal sulcus, supramarginal gyrus and postcentral gyrus, inferior frontal and superior frontal regions is somewhat difficult, since these regions are not known to be specifically involved in reward or feedback processing. Given that the reward participants received in this study was merely artificial in that they were reflected by the gain in chocolate bars summed up on an account, feedback processing might consist of more abstract, higher-level cognitions. For example, the increased activity in these regions could be related to the processing of spatial information of the wheel of fortune (e.g., "what is the relation to the initial speed set and the position of the wheel when it stops?"). Alternatively, changes in cerebral blood flow may have been induced through a heightened state of emotional/autonomic arousal or through attentional processes.

Neuronal networks increasingly active with brand preference in the outcome phase have been commonly linked to feedback processing, bodily perception of pleasurable arousal, and visuo-spatial attention. Participants registered the feedback of winning a more preferred brand with increased visual attention (occipital cortex↑) leading to a positive pleasurable feeling (ventral pallidum↑) accompanied with a heightened state of arousal (ventral pallidum↑, cerebellum↑).

### Limitations

One has to bear in mind that the neural activity pattern found in our study reflects to a certain degree interactions of subjective preference and the experimental task. For example, our participants expected a reward with uncertainty. It has been repeatedly shown that the factor of reward probability partly alters the involvement of the reward network [19,58,59]. A second, and in our view, important factor is whether participants actually receive immediate sensory or material rewards, or delayed symbolic rewards obtained only after conclusion of the experiment. In our study, participants received the won bars of chocolate after the experiment outside the scanner. This is important, considering that partly different brain activations were found for instance in the study by O'Doherty in which participants received differently tasting liquids during the experiment [25], compared with studies in which participants were rewarded with announcements of small money amounts that they received only after the scanning procedure [3,26,60].

This temporal delay of the actual reward receipt may partly explain why we did not observe preference-dependent activity in some brain areas previously indicated by studies exploring reward-related brain activations (e.g., prefrontal cortex or nucleus accumbens, for a review, see [58]). Another striking difference between our and previous studies is that we use differentially preferred incentives of the same product class and same price category. The rewards expected by a participant – and evaluated after the outcome of the wheel-of-fortune game – did not differ significantly in their magnitude of objective (e.g., monetary) value, solely in the magnitude of subjective value. Participants possibly wanted to win each trial and "liked" every won winning trial. The rewards are objectively the same (one bar of chocolate), the only difference being the subjective preference of the reward. Our aim was to identify the neuronal correlates of the subjective, culturally learned preferences that have a modulatory impact on wanting and liking, and influence approach behavior.

Hemodynamic responses in the lateral orbitofrontal cortex and the insula correlated with the subjective preference for the expected gain during the anticipation phase. Neural activity in the OFC is known to correlate with the incentive value of the expected reward [2]. The additional activations found in the insula support the idea that emotions and feelings are evoked in this phase. Some recent studies propose a functional dissociation between the lateral and mesial OFC activation. While the mesial OFC is most strongly involved during anticipation of rewarding stimuli, the lateral OFC seems to be more strongly activated when punishment or deficits are anticipated [61]. However, as winning trials were explicitly separated from losing trials, the pattern of activation is unlikely to reflect engagement in anticipating losses or punishment rather than receiving rewarding stimuli.

## Conclusion

The results of our study clearly demonstrate that neural activation in reward processing structures is modulated by stimuli varying in subjective reward intensity. This modulation was evident in situations where participants anticipated a reward and in situations where participants evaluated a reward. Contrary to the winner-take-all hypothesis [17], neural activity was linearly associated with the subjective brand preference hierarchy, which is in line with studies using objectively varied amounts of money as rewards. Furthermore, distinct brand-preference-modulated areas were identified during anticipation and evaluation phases. When participants anticipate winning a more preferred brand they experience an increased state of wanting. This is characterized by intensified processing of external information and emotional tagging of the incentive stimulus, leading to a state of facilitated action induction. Thus, the pattern of activity may reflect approach behavior in real life situations, such as opting for a particular product on the shopping shelf.

## Authors' contributions

SK and AP contributed equally to this manuscript. SK, AP, AD, VB and LJ designed the experiment. AP programmed the paradigm. SK and AP collected experimental data. SK, AP, AD, VB, LJ analyzed experimental data. AP designed and prepared illustrations. SK, AP, AD, VB, LJ wrote the article.

## Competing interests

The authors declare that they have no competing interests.

## Supplementary Material

Additional file 1 Supplement 1. Short description of the data: Tables of brain structures with linearly modulated activity in the anticipation and outcome phases of losing trials. Click here for file

Additional file 2 Supplement 2. Short description of the data: Tables of main effects of the task for the anticipation phase of winning trials and for the outcome phase of won winning trials. Click here for file
