# Supplementary material for: Individual preferences modulate incentive values: Evidence from functional MRI
Source: Behav Brain Funct. 2008 Nov 25;4:55. doi: 10.1186/1744-9081-4-55 (PMC2633349; doi:10.1186/1744-9081-4-55)
Supplement: Additional file 1 — Supplement 1. Short description of the data: Tables of brain structures with linearly modulated activity in the anticipation and outcome phases of losing trials. [file 1744-9081-4-55-S1.doc]

**Supplement 1**

Table of significantly linearly modulated structures in the anticipation of losing trials. All clusters show a probability of error of *p* < .001 uncorrected for whole-brain multiple comparisons. The coordinates and *t*-values are at the peak voxels in each cluster (coordinates refer to MNI-space).

| Regions | Right/ Left | Cluster Size  (Voxels) | Coordinates | | | t-value |
| --- | --- | --- | --- | --- | --- | --- |
|  |  |  | X | Y | Z |  |
| Increasing linearly with subjective preference | | | | | | |
| Caudate nucleus | R | 28 | 10 | 24 | 10 | 5.32 |
| Caudate nucleus | L | 26 | -12 | 18 | 16 | 4.83 |
| Decreasing linearly with subjective preference | | | | | | |
| Calcarine sulcus | L | 747 | 0 | -90 | 14 | 6.07 |
| Superior occipital cortex | L |  | -10 | -100 | 24 | 4.36 |
| Cuneus | L |  | -6 | -100 | 16 | 5.67 |
| Lingual gyrus | R | 42 | 12 | -62 | -2 | 4.25 |
| Lingual gyrus | R |  | 2 | -68 | -6 | 3.46 |
| Lingual gyrus | R |  | 16 | -54 | -8 | 3.17 |
| Temporal pole superior | R |  | 60 | 8 | -10 | 3.45 |

Table of significantly linearly modulated structures in the outcome of lost losing trials. All clusters show a probability of error of *p* < .001 uncorrected for whole-brain multiple comparisons. The coordinates and *t*-values are at the peak voxels in each cluster (coordinates refer to MNI-space).

| Regions | Right/ Left | Cluster Size  (Voxels) | Coordinates | | | t-value |
| --- | --- | --- | --- | --- | --- | --- |
|  |  |  | X | Y | Z |  |
| Increasing linearly with subjective preference | | | | | | |
| Inferior frontal operculum | R | 19 | 24 | 8 | 14 | 4.98 |
| Posterior cingulum | L | 20 | -18 | -42 | 20 | 4.85 |
| Decreasing linearly with subjective preference | | | | | | |
| Middle frontal gyrus | L | 26 | -28 | 22 | 44 | 4.85 |
