# Supplementary material for: Individual preferences modulate incentive values: Evidence from functional MRI
Source: Behav Brain Funct. 2008 Nov 25;4:55. doi: 10.1186/1744-9081-4-55 (PMC2633349; doi:10.1186/1744-9081-4-55)
Supplement: Additional file 2 — Supplement 2. Short description of the data: Tables of main effects of the task for the anticipation phase of winning trials and for the outcome phase of won winning trials. [file 1744-9081-4-55-S2.doc]

**Supplement 2**

Table of Main effects of the anticipation phase of winning trials (WA). All clusters show a probability of error of p < .001 uncorrected for whole-brain multiple comparisons. The coordinates and *t*-values are at the peak voxels in each cluster (coordinates refer to MNI-space).

| Regions | Right/ Left | Cluster Size  (Voxels) | Coordinates | | | t-value |
| --- | --- | --- | --- | --- | --- | --- |
|  |  |  | X | Y | Z |  |
| Lateral occipital Cortex | R | 5933 | 26 | -94 | -10 | 19.79 |
| Lateral occipital Cortex | L | 4151 | -30 | -96 | 8 | 13.97 |
| Thalamus | L | 291 | -24 | -30 | -4 | 10.66 |
| Thalamus | R | 378 | 22 | -28 | -2 | 9.49 |
| Caudate | L | 58 | -16 | 28 | -4 | 5.33 |
| Caudate | L | 20 | -22 | -8 | 32 | 5.21 |
| Lateral ventricle / Caudate | R | 67 | 4 | 24 | 2 | 5.10 |
| Brainstem | R | 22 | 16 | -28 | -30 | 4.57 |
| Lateral orbitofrontal Cortex | R | 467 | 32 | 22 | -26 | 6.37 |
| Lateral orbitofrontal cortex | R | 40 | 48 | 46 | -16 | 5.11 |
| Inferior frontal gyrus | L | 491 | -58 | 16 | 4 | 5.37 |
| Inferior frontal gyrus | R | 26 | 60 | 22 | 26 | 4.55 |
| Superior frontal gyrus | R | 14 | 30 | 4 | 68 | 4.06 |
| Insula | R | 11 | 44 | -6 | 22 | 4.22 |
| Premotor cortex | L | 43 | -22 | -8 | 60 | 4.42 |
| Premotor cortex | L | 10 | -40 | -4 | 48 | 4.02 |
| Precentral gyrus | R | 116 | 38 | -10 | 36 | 6.00 |
| Postcentral gyrus | L | 10 | -60 | -6 | 38 | 4.04 |
| Cerebellum | R | 23 | 12 | -78 | -46 | 4.49 |
| Vermis | R | 15 | 2 | -34 | -32 | 4.17 |
| White matter / posterior corona radiata | L | 12 | -28 | -30 | 28 | 4.29 |

Table of Main effects of the outcome phase of won winning trials (WOW). All clusters show a probability of error of p < .001 uncorrected for whole-brain multiple comparisons. The coordinates and *t*-values are at the peak voxels in each cluster (coordinates refer to MNI-space).

| Regions | Right/ Left | Cluster Size  (Voxels) | Coordinates | | | t-value |
| --- | --- | --- | --- | --- | --- | --- |
|  |  |  | X | Y | Z |  |
| Occipital cortex | R/L | 14927 | -8 | -86 | -12 | 11.34 |
| Parieto-occipital cortex | L | 2119 | -48 | -60 | 30 | 9.45 |
| Precuneus | R | 13 | 2 | -62 | 62 | 4.51 |
| Caudate | L | 236 | -14 | 0 | 28 | 6.08 |
| White matter / Caudate | L | 12 | -12 | 14 | 22 | 5.23 |
| Caudate | R | 27 | 14 | 14 | 20 | 5.22 |
| Hippocampus | R | 13 | 28 | -32 | 8 | 4.91 |
| Parahippocampal gyrus | L | 17 | -34 | -12 | -24 | 5.01 |
| Middle frontal gyrus | L | 174 | -38 | 8 | 44 | 6.79 |
| Middle frontal gyrus | R | 211 | 40 | 26 | 24 | 6.07 |
| Inferior frontal gyrus | L | 526 | -50 | 28 | 0 | 6.29 |
| Superior frontal gyrus | R | 119 | 2 | 28 | 62 | 6.15 |
| Frontal Pole | R | 48 | 44 | 52 | -10 | 5.74 |
| Middle frontal gyrus | R | 73 | 42 | 18 | 48 | 5.68 |
| Frontal Pole | L | 33 | -2 | 62 | 26 | 5.63 |
| Orbitofrontal cortex | L | 46 | -34 | 22 | -26 | 5.50 |
| Frontal pole | L | 13 | -6 | 64 | -2 | 5.38 |
| Dorsomedial prefrontal cortex | R | 83 | 18 | 48 | 48 | 5.28 |
| Premotor cortex | L | 158 | -10 | 14 | 68 | 6.36 |
| Premotor cortex | R | 20 | 38 | -20 | 68 | 4.89 |
| Posterior cingulate gyrus | R | 722 | 4 | -46 | 34 | 5.43 |
| Middle temporal gyrus | R | 2422 | 68 | -32 | -8 | 9.20 |
| Temporal pole | R | 113 | 52 | 16 | -22 | 6.40 |
| Temporal Pole | L | 12 | -54 | 4 | -26 | 5.54 |
| Middle temporal gyrus | R | 18 | 62 | -4 | -26 | 5.18 |
| Temporal pole | L | 16 | -40 | 16 | -34 | 5.07 |
| Temporal pole | R | 33 | 38 | 18 | -42 | 4.33 |
| Temporal fusiform cortex | L | 10 | -44 | -40 | -22 | 4.92 |
| Precentral gyrus | R | 69 | 64 | -2 | 10 | 4.62 |
| Postcentral gyrus | R | 327 | 10 | -38 | 74 | 7.06 |
| Postcentral gyrus | L | 30 | -12 | -42 | 74 | 5.46 |
| Parietal operculum | R | 55 | 46 | -8 | 18 | 6.46 |
| Parietal operculum | L | 130 | -26 | -30 | 20 | 6.06 |
| Parietal operculum | R | 45 | 40 | -26 | 26 | 5.40 |
| Cerebellum | R | 29 | 28 | -76 | -50 | 5.41 |
| Cerebellum | - | 27 | 0 | -72 | -46 | 5.30 |
| Cerebellar tonsil | R | 12 | 10 | -56 | -36 | 3.92 |
| White matter/ putamen | R | 108 | 32 | -2 | 24 | 5.07 |
| Genu of corpus callosum | L | 191 | -2 | 24 | 8 | 6.58 |
| Intraparietal Gyrus | R | 46 | 24 | -54 | 36 | 4.54 |
| White matter / premotor cortex | R | 11 | 24 | -22 | 44 | 4.31 |
| Lateral ventricle | R | 13 | 6 | -30 | 16 | 4.25 |
